# Incidental Discovery of a Morgagni Hernia in a 72-Year-Old Woman After COVID-19 Pneumonia: A Case Report

**DOI:** 10.7759/cureus.58799

**Published:** 2024-04-23

**Authors:** Mouhsin Ibba, Razouq Boujemaa, Hicham Fenane, Yassine Msougar

**Affiliations:** 1 Thoracic Surgery Department, Mohammed VI University Hospital, Marrakesh, MAR

**Keywords:** covid-19, sars-cov-2, diaphragm treatment, congential diaphragmatic hernia, lateral thoracotomy, adult morgagni hernia

## Abstract

Morgagni hernia is a rare condition characterized by a congenital retrosternal defect of the diaphragm, leading to the protrusion of abdominal organs into the thoracic cavity. Here, we report the case of a 72-year-old woman with a Morgagni hernia incidentally discovered during evaluation for persistent dyspnea following COVID-19 pneumonia. The diagnosis was made by imaging, including a chest X-ray and a thoracic CT scan, which showed an ascent of the transverse colon and omentum through an anterior retrosternal defect. Surgical exploration via right posterolateral thoracotomy revealed an anterior diaphragmatic hernia with a small defect containing the greater omentum and transverse colon, which was repaired by resecting the hernia sac and closing the diaphragmatic defect by fixing the anterior rim of the diaphragm to the retrosternal fascia with interrupted silk sutures. Postoperative recovery was uneventful, and follow-up examinations revealed no abnormalities on chest X-rays obtained at one, three, and six months. This case highlights the incidental discovery and successful surgical management of a Morgagni hernia in an elderly patient through a thoracic approach.

## Introduction

Congenital diaphragmatic hernia is a rare condition resulting from a congenital defect of the diaphragm, leading to the protrusion of abdominal viscera into the thoracic cavity. It can occur as an isolated anomaly (in 40% of cases) or be associated with other malformations. The hernia is typically located posterolaterally in 85% of cases (Bochdalek hernia), and less commonly anterolaterally (Morgagni and Larrey hernia), leading to respiratory and cardiac complications [1,2]. Here, we report the case of a 72-year-old female presenting a Morgagni hernia discovered incidentally on evaluation for persistent dyspnea following COVID-19 pneumonia.

## Case presentation

**Figure 1:**
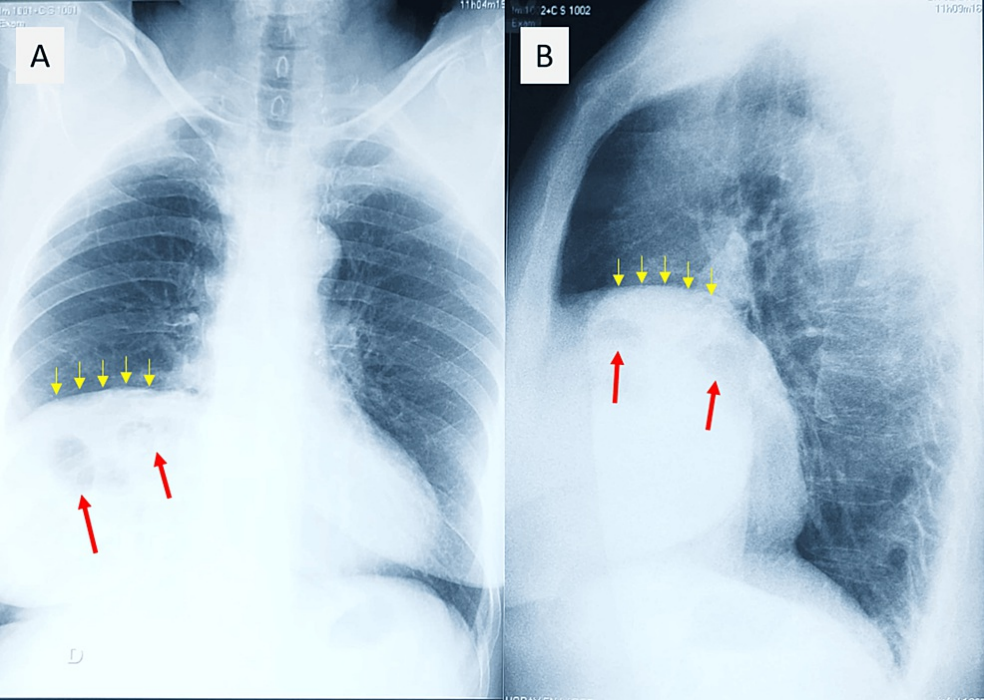
Chest X-ray posteroanterior (A) and lateral (B) views showing an elevation of the right diaphragmatic dome (yellow arrows) with intrathoracic air-fluid images (red arrows).

**Figure 2:**
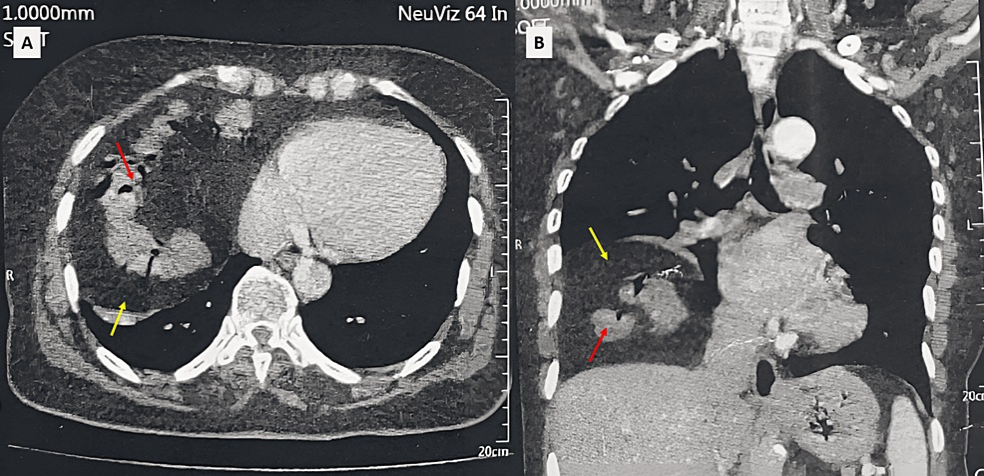
Axial (A) and coronal (B) sections of chest CT scan showing an ascent of a portion of the transverse colon (red arrow) and omentum (yellow arrow) through an anterior retrosternal defect.

A 72-year-old woman had a history of moderate COVID-19 pneumonia episodes occurring six months before admission, without a history of intensive care unit stay. The diagnosis of SARS-CoV-2 infection was confirmed by a reverse transcription polymerase chain reaction test, and no imaging was performed at that time. She experienced persistent dyspnea despite the resolution of other symptoms with medical treatment. Physical clinical examination did not reveal significant abnormalities. She denied fevers, weight loss, chest pain, or hemoptysis. The blood tests demonstrated normal full blood counts and coagulation profile. The electrocardiogram did not reveal any abnormality. Chest X-ray showed an elevation of the right diaphragmatic dome with intrathoracic air-fluid images (Figure 1). A thoracic CT scan revealed an ascent of a portion of the transverse colon and omentum through an anterior retrosternal defect (Figure 2). Thus, the diagnosis of Morgagni hernia was made. The patient had no history of known congenital malformations or preexisting abdominal symptoms.

**Figure 3:**
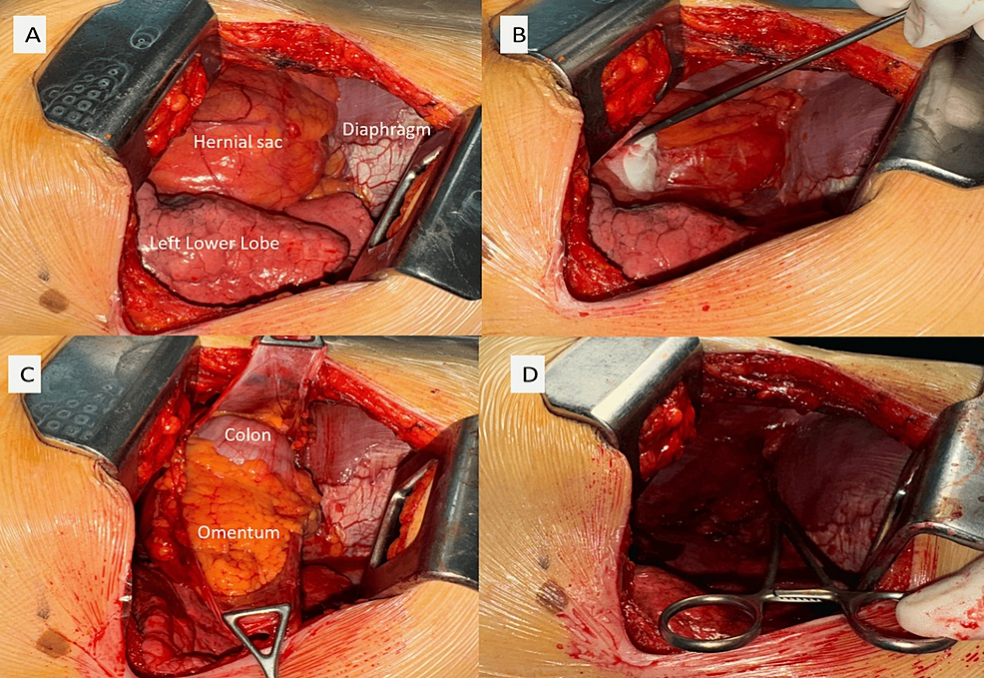
Intraoperative images showing the different stages of hernia repair. Protrusion of the hernia sac into the thoracic cavity through a 3 cm defect (A and B). Opening of the sac and reintroduction of its contents into the abdomen followed by closure of the defect (C and D).

Following a transthoracic approach, surgical exploration by right posterolateral thoracotomy revealed an anterior diaphragmatic hernia with a small defect measuring 3 cm containing the greater omentum and transverse colon, adherent to the hernia sac. The hernia sac was dissected and resected, and its contents were reintroduced into the abdomen. The diaphragmatic defect was closed by fixing the anterior rim of the diaphragm to the retrosternal fascia with 0-gauge interrupted silk sutures (Figure 3).

**Figure 4:**
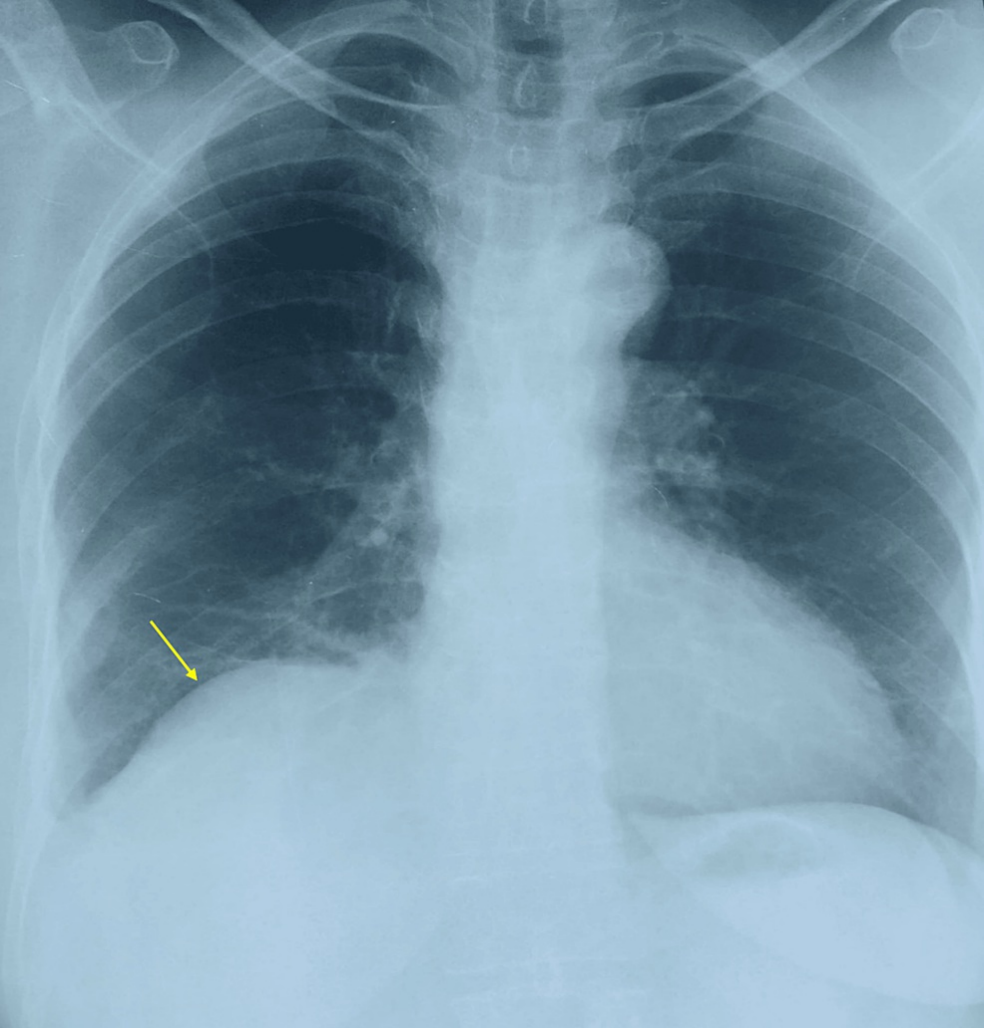
Postoperative chest X-ray showing normalization of the appearance of the diaphragmatic dome (yellow arrow) and disappearance of air-fluid images.

Immediate postoperative recovery was uneventful, and the patient was discharged on the second postoperative day. Follow-up examinations showed no abnormalities, and chest X-rays obtained at one, three, and six months revealed normalization of the appearance of the diaphragmatic dome, with the disappearance of air-fluid images (Figure 4).

## Discussion

Congenital diaphragmatic hernias are rare, occurring in 27-47% of cases, and are often associated with other malformations (pulmonary, abdominal, cardiac, genitourinary, skeletal, central nervous system) and chromosomal anomalies (Down’s syndrome) [3]. They are typically diagnosed neonatally [2]. However, when they present in adulthood, they are usually incidentally discovered without clinical significance [1]. They include hernias of the diaphragmatic domes, known as Bochdalek hernias when they are left posterolateral (but can also occur on the right), and anterior or retrosternal-costoxiphoid hernias, which are rarer (1-6% of diaphragmatic hernias). Right sternocostal hiatus hernias are called Morgagni hernias, described by Morgagni in 1761, and those on the left sternocostal hiatus are called Larrey’s slit hernias. If the opening is large enough to include both hiatuses, they are referred to as Morgagni-Larrey hernias. Usually, there is no clinical distinction between the two defects, and thus the term Morgagni hernia is commonly used [1,4].

Morgagni hernias are generally asymptomatic but may occasionally manifest with nonspecific epigastric or retrosternal discomfort, or more rarely with respiratory symptoms. The nonspecific nature of the symptoms can lead to diagnostic delays. Complications such as strangulation of the herniated colon or stomach due to constriction are rare [2,5]. Diagnosis can be made on chest radiography showing an opacity containing gas shadows above the right dome. CT and, rarely, MRI reveal fatty masses in the cardiophrenic angle, differentiating them from physiological pericardial fat by the diaphragmatic discontinuity and displaced omental vessels [2]. The differential diagnosis of congenital diaphragmatic hernias includes anterior mediastinal tumors, pleuropericardial cysts, incomplete pneumothorax, pulmonary atelectasis, or simple lipoma, as well as diaphragmatic eventrations [1,2].

Surgical treatment is always recommended for Morgagni hernias due to the risk of severe complications, except in cases of asymptomatic hernias in elderly and frail patients [6]. The goal is to repair the diaphragmatic dome while preserving pulmonary compliance as much as possible, reintegrating gastrointestinal elements into the abdomen, and preventing complications [7]. This can be done via laparoscopic or thoracoscopic approaches or even robotic surgery, safe minimally invasive approaches with low morbidity and shorter hospital stays [2,8], or via open surgery (laparotomy or thoracotomy) [3]. The surgical procedure involves reintroducing hernial contents, hernial sac resection is desirable but can be challenging on the left side due to adhesions to the pericardium, followed by the closure of the anterior diaphragmatic edge to the sternum or anterior abdominal wall with separate sutures or nonabsorbable thread. In cases of severe diaphragmatic hypoplasia, diaphragmatic reconstruction with a muscle flap (latissimus dorsi) may be necessary. Aspirative drainage of the thoracic cavity may be necessary. Diaphragmatic suture is often accompanied, whenever possible, by additional reinforcement with mesh [8].

Postoperative complications are rare in simple diaphragmatic hernias, and outcomes are generally favorable. However, mortality is 20-32% in cases of emergency intervention for acute complications [2].

## Conclusions

This case highlights a remarkable asymptomatic evolution of a Morgagni hernia in a 72-year-old patient and provides valuable insights for healthcare practitioners regarding the diagnostic and therapeutic management of such hernias in elderly individuals through a thoracic approach. By emphasizing the importance of clinical vigilance and prompt surgical evaluation, this observation contributes to our understanding of the complexities in managing diaphragmatic hernias in the elderly population.
